# Epithelioid Hemangioendothelioma as a Dangerous, Easy to Miss, and Nearly Impossible to Clinically Diagnose Condition: Case Report

**DOI:** 10.2196/52493

**Published:** 2024-08-28

**Authors:** Kayd Pulsipher, Samantha Mills, Blair Harris, Rene Bermudez, Muammar Arida, Jonathan Crane

**Affiliations:** 1 Department of Dermatology Campbell University Sampson Regional Center Clinton, NC United States; 2 Campbell University School of Osteopathic Medicine Lillington, NC United States

**Keywords:** epithelioid hemangioendothelioma, EHE, vascular tumor, tumor, vascular, blood vessel, cutaneous, skin, lesion, histopathology, case report, metastatic, dermatology, dermatological, diagnose, diagnosis, rare cancer, oncology

## Abstract

Epithelioid hemangioendothelioma (EHE) is a rare vascular tumor with metastatic potential. EHE can have single- or multiorgan involvement, with presentations ranging from asymptomatic disease to pain and systemic symptoms. The extremely heterogeneous clinical presentation and disease progression complicates EHE diagnosis and management. We present the case of a 24-year-old woman with two periauricular erythematous papules, leading to the discovery of metastatic EHE through routine biopsy, despite a noncontributory medical history. Histology revealed the dermal proliferation of epithelioid cells and vacuoles containing red blood cells. Immunohistochemistry markers consistent with EHE solidified the diagnosis. Although extremely rare, prompt diagnosis of EHE is essential for informed decision-making and favorable outcomes. Key clinical and histopathological findings are highlighted to aid dermatologists in diagnosing and managing this uncommon condition.

## Introduction

Epithelioid hemangioendothelioma (EHE) is an extremely rare cancer, accounting for less than 1% of all vascular tumors, demonstrating features between those of hemangioma and angiosarcoma [1]. Although first described by Dail and Liebow in 1975, the term EHE was only first proposed by Weiss and Enzinger in 1982 [1]. These tumors can occur at any age, with 38 years being the median age at diagnosis [2]. The most common presenting symptom is pain, along with less commonly reported symptoms such as cough, palpable mass, or fatigue. Nearly one-third of patients with EHE are asymptomatic and tumors are discovered incidentally [2]. While likely endothelial in origin, EHE is extremely heterogeneous in presentation and prognosis, complicating diagnosis and clinical decisions [3]. EHE can occur nearly anywhere in the body. Primary cutaneous EHE is rare and should prompt suspicion of metastatic disease, especially if multifocal in the skin [4]. Owing to their rarity and similarities to other diagnoses, cutaneous EHE lesions are commonly misdiagnosed [5]. Previous studies suggest the diagnosis of strictly cutaneous EHE incurs a 17% mortality rate at 3 years, highlighting its relatively aggressive nature [6]. It is paramount for dermatologists and dermatopathologists to be aware of EHE and its defining characteristics to minimize the risk of missing this crucial diagnosis.

We report a case of two periauricular lesions with dermal proliferation consistent with EHE, leading to the discovery of underlying metastatic EHE with pulmonary and hepatic involvement in a 24-year-old woman. The aim of presenting this case is to enhance understanding of EHE, an uncommon cancer that is not well studied.

## Case Report

**Figure 1:**
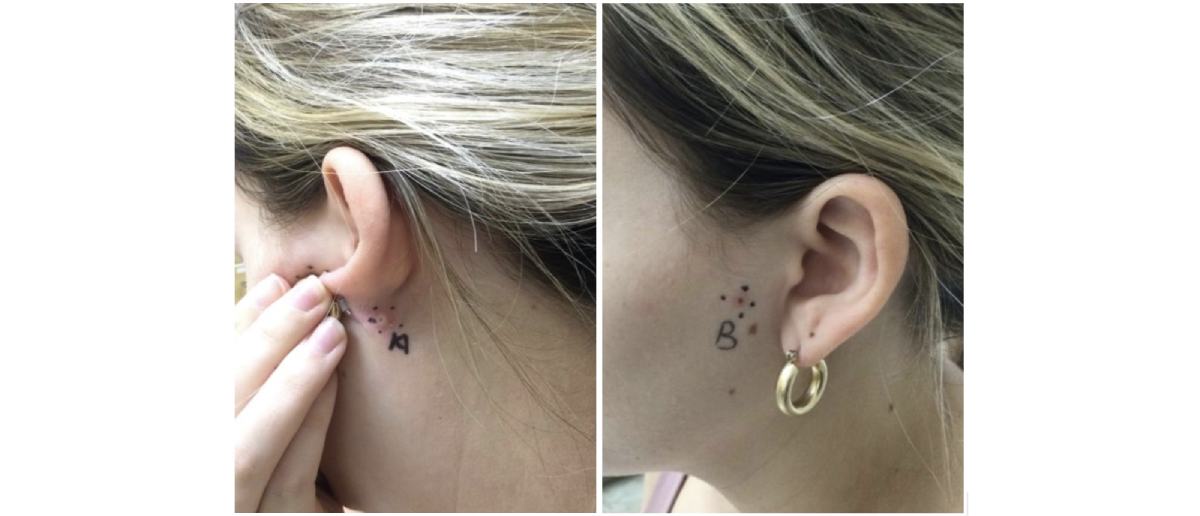
A 3-mm umbilicated, skin-colored papule on the post auricular neck (left) and a 2-mm hyperpigmented papule with surrounding erythema on the preauricular cheek (right).

A 24-year-old woman presented to our dermatology clinic with a left posterior auricular papule and left preauricular papule present for 8 and 4 months, respectively (Figure 1). The patient had no significant medical or social history, including no tobacco or heavy alcohol use. Both lesions were painful and progressively enlarging. The patient denied any other symptoms.

**Figure 2:**
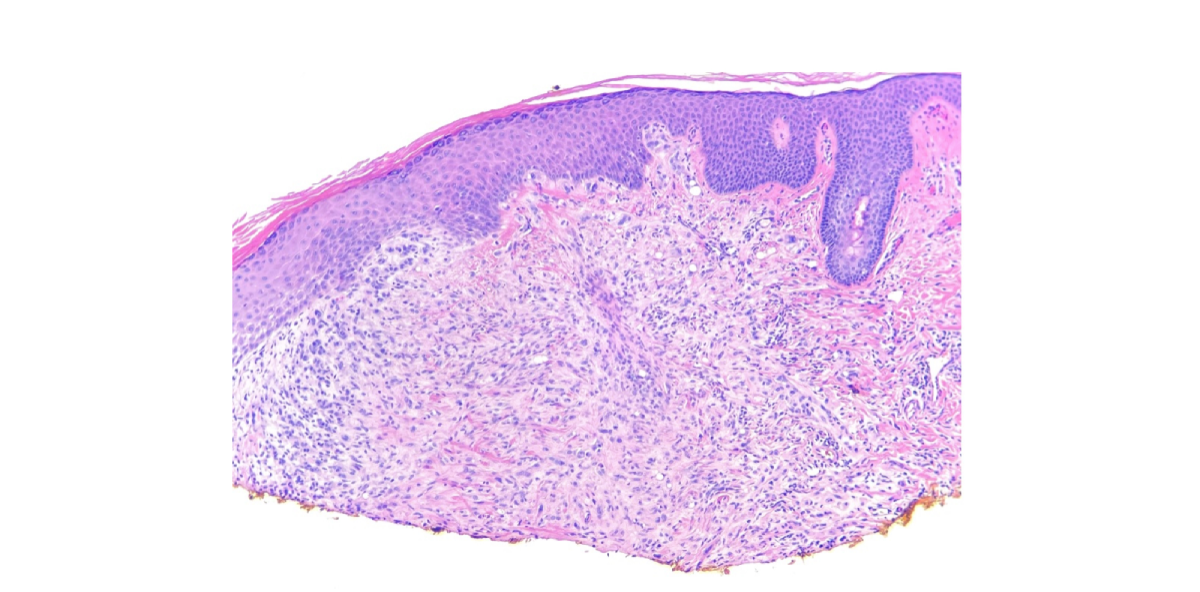
Dense proliferation of dermal epitheliod cells with no attachment to the epidermis.

**Figure 3:**
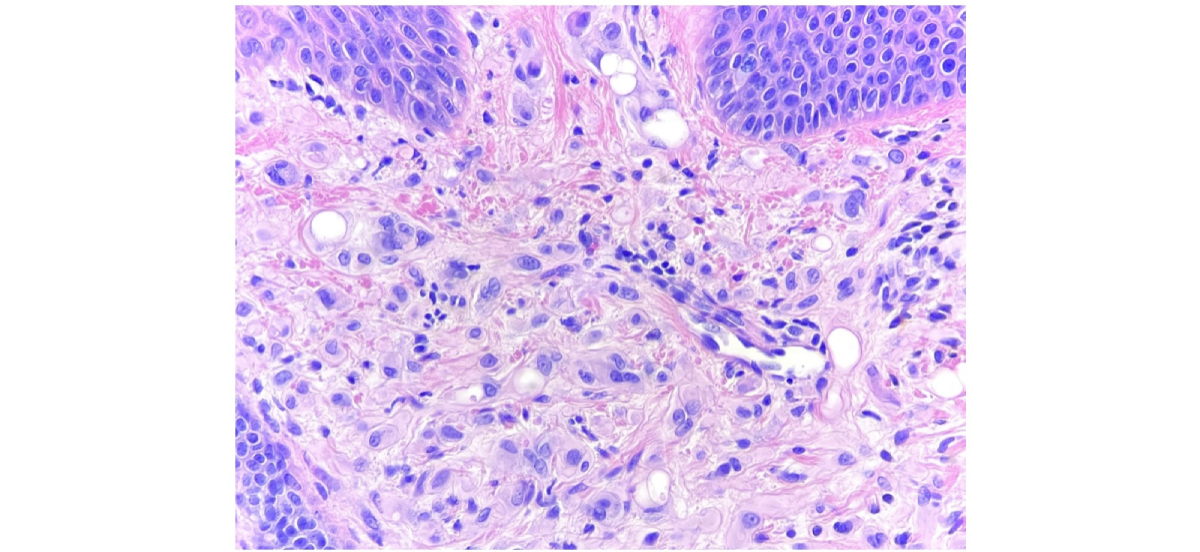
Dermal epithelioid cells with eosinophilic cytoplasm and red blood cells in vacuolations identifying them as vascular spaces.

Shave biopsy was taken of both lesions. The histology of both lesions demonstrated cellular dermal proliferations of epithelioid cells with the eosinophilic cytoplasm arranged in cords within a myxohyaline stroma (Figure 2). Subtle vacuoles containing red blood cells were present within some of the cells (Figure 3). Histological and immunohistochemical findings were consistent with the diagnosis of EHE (Table 1).

Due to multifocal cutaneous disease, there was high clinical suspicion of metastatic disease. Our patient was referred to medical and surgical oncology for further evaluation, and computed tomography (CT) scans of the head, neck, chest, abdomen, and pelvis were performed. Innumerable bilateral pulmonary nodules, a 1.8-cm hypoattenuated hepatic lesion, and prominent bilateral axillary lymph nodes were noted, all consistent with metastatic disease. After seeking multiple opinions from oncology, our patient elected the watchful waiting approach. Serial CT scans every 3 months were recommended to monitor disease progression.

**Table 1:** Immunohistochemistry patterns characteristic of epithelioid hemangioendothelioma (EHE) and the staining results of the patient.

EHE immunohistochemistry markers	Result in patient
ERG	Positive (diffuse)
CD31	Positive (diffuse)
α-SMA^a^	Negative
FVIII Ag^b^	—^c^
CAMTA1/TFE3	Positive (diffuse)
Focal cytokeratin (<30%^d^)	Positive
SOX10	Negative

^a^SMA: smooth muscle actin.

^b^FVIII Ag: factor VIII-related antigen.

^c^Not tested.

^d^Percentages refer to the estimated prevalence in EHE tumors [3,6,7].

## Ethical Considerations

The patient provided consent to publish information regarding her case, including photographs and relevant findings. Identifiable patient information has been appropriately masked or omitted to comply with ethical standards and patient privacy.

## Discussion

### Prior Reports of EHE

Literature pertaining to EHE is limited with case reports and case series comprising the majority. This can largely be attributed to the low prevalence of EHE, reported as approximately 1 in 1 million [1]. Sites of primary and metastatic involvement in EHE most commonly involve the liver, lung, and bone; however, the disease has been reported in nearly every part of the body. When cutaneous EHE is discovered, it typically represents metastatic disease rather than primary malignancy. The appearance, location, and characteristics of cutaneous EHE vary immensely from case to case, with no clear consensus available [4,7,8]. The extreme heterogeneity of this disease complicates detection and diagnosis [2].

Histopathology and immunohistochemistry are often crucial for diagnosis of cutaneous disease. Histologically, tumors typically show circumcised nodules with an overlying acanthotic epidermis. A mixture of pleomorphic spindle and epithelioid cells with sharply eosinophilic cytoplasm will be present, typically embedded in a myxoid or hyaline matrix [8]. Cells typically stain positive for CD31, CD34, factor VIII-related antigen, α-smooth muscle actin, and cytokeratin [6,7]. When unable to be clearly differentiated from other vascular tumors, the presence of the *WWTR1-CAMTA1* translocation can aid the diagnosis of EHE [3]. This translocation dysregulates the Hippo pathway, promoting cancer proliferation and survival [9].

The prognosis of strictly cutaneous EHE is not readily available. In a small case series of 30 patients with cutaneous EHE, at 36 months follow-up, 21% of cases had metastatic disease, 13% had local recurrence, and 17% had died from the disease [6]. In all cases of EHE irrespective of site, 1-year overall survival is 90% with a 5-year overall survival of 73% [2].

Given the low prevalence of EHE, no randomized clinical trials exist regarding the optimal treatment strategy [7]. Patients with cutaneous EHE should receive additional imaging to evaluate for metastatic disease. When no metastatic disease is found, the treatment is surgical resection [3]. A variety of treatments such as cytotoxic chemotherapy, immunotherapy, targeted therapies, and organ transplantation have been used for metastatic disease (Table 2). With reports of spontaneous disease regression [10], watchful waiting can also be proposed as a reasonable course following EHE diagnosis, especially if the nature of the disease is not yet understood or the risks of treatment outweigh benefits.

**Table 2:** Possible treatment options for epithelioid hemangioendothelioma based on retrospective studies of tumor involvement and case outcomes [9].

Involvement and considerations			Treatment
**Unifocal involvement**			
	R0^a^ margins	Surgical resection (70%-80% cure rate)	
	R1^b^ margins	Surgical resection ± radiation therapy	
	Severe morbidity or R0/R1 not possible	Radiation therapy/ ablative procedure/ isolated limb perfusion	
	Not surgical candidate (comorbidities or technical challenges)	Active surveillance	
**Locoregional**			
	Resection possible	Surgical resection ± radiation therapy	
	Asymptomatic	Active surveillance	
	Symptomatic (surgery not possible)	Radiation therapy/ ablative procedure/ isolated limb perfusion	
**Systemic**			
	Resection possible	Surgical resection ± radiation therapy	
	Asymptomatic	Active surveillance	
	Symptomatic (systemic) or serosal effusion	Systemic therapy (limited evidence)	
	Organ involvement	Surgical resection/transplant (unresectable)	

^a^R0: microscopic negative margins.

^b^R1: gross negative margins.

### Conclusion

The heterogeneity of EHE is also demonstrated in its variable course; EHE can be unpredictable, at times being indolent and at other times very aggressive [7]. Given the uncertain course of the disease, joint decision-making between the patient and physician is necessary. Active surveillance includes monitoring progression, and the decision to treat with radiation or surgery often follows once the nature of the tumor is better understood [9]. Systemic treatments have been recorded, but not enough data are currently available to determine a standard approach [9]. Regardless of the course of management, close follow-up for local recurrence and metastatic disease is essential. Future studies should focus on early detection and a standardized approach for the treatment EHE.

## Abbreviations

CT: computed tomography
EHE: epithelioid hemangioendothelioma

## References

[1] Sardaro A, Bardoscia L, Petruzzelli MF, Portaluri M (2014). Epithelioid hemangioendothelioma: an overview and update on a rare vascular tumor. Oncol Rev.

[2] Lau K, Massad M, Pollak C, Rubin C, Yeh J, Wang J, Edelman G, Yeh J, Prasad S, Weinberg G (2011). Clinical patterns and outcome in epithelioid hemangioendothelioma with or without pulmonary involvement: insights from an internet registry in the study of a rare cancer. Chest.

[3] Witte S, Weidema M, Kaal S, Versleijen-Jonkers Y, Flucke U, van der Graaf W, Desar I (2021). The heterogeneity of epithelioid hemangioendothelioma (EHE): a case series and review of the literature with emphasis on treatment options. Semin Oncol.

[4] Snyder ML, Lyle S, Dinh T, Stead J, Kannler C (2020). Primary cutaneous epithelioid hemangioendothelioma with lymph node metastasis. JAAD Case Rep.

[5] Weiss SW, Enzinger FM (1982). Epithelioid hemangioendothelioma: a vascular tumor often mistaken for a carcinoma. Cancer.

[6] Mentzel T, Beham A, Calonje E, Katenkamp D, Fletcher CD (1997). Epithelioid hemangioendothelioma of skin and soft tissues: clinicopathologic and immunohistochemical study of 30 cases. Am J Surg Pathol.

[7] Rosenberg A, Agulnik M (2018). Epithelioid hemangioendothelioma: update on diagnosis and treatment. Curr Treat Options Oncol.

[8] Quante M, Patel NK, Hill S, Merchant W, Courtauld E, Newman P, McKee PH (1998). Epithelioid hemangioendothelioma presenting in the skin: a clinicopathologic study of eight cases. Am J Dermatopathol.

[9] Stacchiotti S, Miah A, Frezza A, Messiou C, Morosi C, Caraceni A, Antonescu C, Bajpai J, Baldini E, Bauer S, Biagini R, Bielack S, Blay J, Bonvalot S, Boukovinas I, Bovee J, Boye K, Brodowicz T, Callegaro D, De Alava E, Deoras-Sutliff M, Dufresne A, Eriksson M, Errani C, Fedenko A, Ferraresi V, Ferrari A, Fletcher C, Garcia Del Muro X, Gelderblom H, Gladdy R, Gouin F, Grignani G, Gutkovich J, Haas R, Hindi N, Hohenberger P, Huang P, Joensuu H, Jones R, Jungels C, Kasper B, Kawai A, Le Cesne A, Le Grange F, Leithner A, Leonard H, Lopez Pousa A, Martin Broto J, Merimsky O, Merriam P, Miceli R, Mir O, Molinari M, Montemurro M, Oldani G, Palmerini E, Pantaleo M, Patel S, Piperno-Neumann S, Raut C, Ravi V, Razak A, Reichardt P, Rubin B, Rutkowski P, Safwat A, Sangalli C, Sapisochin G, Sbaraglia M, Scheipl S, Schöffski P, Strauss D, Strauss S, Sundby Hall K, Tap W, Trama A, Tweddle A, van der Graaf W, Van De Sande M, Van Houdt W, van Oortmerssen G, Wagner A, Wartenberg M, Wood J, Zaffaroni N, Zimmermann C, Casali P, Dei Tos A, Gronchi A (2021). Epithelioid hemangioendothelioma, an ultra-rare cancer: a consensus paper from the community of experts. ESMO Open.

[10] Kitaichi M, Nagai S, Nishimura K, Itoh H, Asamoto H, Izumi T, Dail D (1998). Pulmonary epithelioid haemangioendothelioma in 21 patients, including three with partial spontaneous regression. Eur Respir J.

